# Medial plantar artery perforator (MPAP) flap is an ideal option for reconstruction of complex soft tissue defect in the finger: Clinical experience from 11 cases

**DOI:** 10.3389/fsurg.2022.934173

**Published:** 2022-07-26

**Authors:** Xiang Xu, Cheng Wang, Zhenbing Chen, Jin Li

**Affiliations:** Department of Hand Surgery, Union Hospital, Tongji Medical College, Huazhong University of Science and Technology, Wuhan, China

**Keywords:** medial plantar flap, perforator flap, complex soft tissue defects, finger reconstruction, lobulated flap

## Abstract

**Introduction:**

Soft tissue defects of fingers are common in reconstructive plastic surgery, and reconstruction of the defects remains challenging for plastic surgeons. In our study, we reported our experience in finger reconstruction with a medial plantar artery perforator (MPAP) flap, especially using a lobulated MPAP flap for the complex multifinger soft defect.

**Patients and methods:**

From the period April 2012 to October 2018, 11 patients (9 males and 2 females) with an average age of 44 years old (ranging from 11 to 58) received finger reconstruction with a free MPAP flap. In total, 11 flaps (8 single-lobulated flaps and 3 two-lobulated flaps) were raised from the ipsilateral or contralateral instep area. Trauma and scar contracture caused hand soft tissue loss in all cases.

**Results:**

The sizes of the flaps ranged from 2×3 to 5×7.5 cm^2^. All flaps survived intact with no complications. One donor site was closed primarily, and other donor sites were covered with a full-thickness skin graft. The mean follow-up time was 6 months (ranging from 3 to 8 months). During the follow-up period, the patients were satisfied with their appearance without any traces of flap plastic surgery.

**Conclusion:**

The MPAP flap is a reliable and acceptable option for the reconstruction of complex soft tissue defects in the finger. Depending on the two branches of the medial plantar artery, the use of the lobulated MPAP flap holds promise in the treatment of multifinger soft tissue defects.

## Introduction

In emergency departments, hand injuries with exposed tendons, nerves, blood vessels, and bones can be commonly seen (1). Due to the durability, mobility, and sensory functions of fingers, the candidate donors are required to have similar properties, texture, thickness, and sensitivity (1, 2). Meanwhile, less significant morbidity of the donor site and complication rate should be considered in choosing surgical methods (3–5). It is challenging for surgeons to repair wounds, restore appearance, and reconstruct the functions of fingers.

Various surgical methods reconstructing and repairing finger soft defects were reported, such as V-Y advancement flaps, cross-finger flaps, digital artery island flaps, and thenar flaps (2, 3, 6, 7). The methods using adjacent and similar skin and soft tissues brought satisfying outcomes, but deficiencies and limitations still exist, including limited advancement distance, insufficient dimensions, two-stage surgeries, and donor site morbidity (7, 8). Recently, more and more free artery perforator flaps were applied to the treatment of finger and palm skin defects, like the anterolateral thigh flap, lateral arm flap, and radial artery perforator flap (9–11). However, the thickness and the donor area morbidity of free artery perforator flaps also hampered their prevalence. To correct these deficiencies, an ideal flap for restoring defects in hands should be thin and small enough with minimal donor site morbidity, and “like with like” skin color and texture are demanding.

Taylor and Hopson first described and used medial plantar artery flaps to repair knee defects in the year 1975 (12). Further, medial plantar artery flaps were also used in the reconstruction of ankle, foot, plantar, hand, and finger (13–16). With proper thickness, similar anatomical structure, sufficient flap size, and low donor site morbidity, the medial plantar artery flap was considered an ideal option for finger soft defect reconstruction. The clinical applications of medial plantar artery flaps (including instep flap and medial pedis flap) in finger defect reconstruction were reported (17, 18). With the development of artery perforator flaps, the medial plantar artery perforator (MPAP) flap was originally introduced by Koshima et al. and used in restoring finger defects (4, 19). The MPAP can be harvested from hairless non-weight-bearing medial plantar regions of the foot and defined as a perforator flap based on the superficial or deep branch of the medial plantar artery (MPA). Based on the arterial perforator, the medial plantar artery perforator flap can be designed as lobulated flaps catering to complex or multiple skin defects of the finger. In this article, we report our experience using the MPAP flap for finger defect reconstruction. Moreover, we present operative techniques of the lobulated medial plantar artery flap and evaluate its feasibility in clinical routine.

## Patients and methods

From April 2012 to October 2018, 11 patients, namely 9 males and 2 females with an average age of 44 years old (ranging from 11 to 58), underwent hand or finger reconstruction surgeries with the MPAP flap. This study followed the ethical committee guidelines of our institution, and the protocol was developed in accordance with the ethical standards of the Helsinki Declaration of 1975 and all subsequent revisions. Written informed consent was obtained from all the patients. Trauma and scar contracture caused hand soft tissue loss in all cases. Skin grafting, primary closure, and traditional repair techniques (V-Y advancement flaps, cross-finger flaps, digital artery island flaps, and thenar flaps) are not effective enough in finger reconstruction. Of the 11 patients, 8 accepted a single-lobulated medial plantar artery perforator flap with perforator from the medial branch of the deep branch of the medial plantar artery (MPA) and the remaining three accepted a two-lobulated medial plantar artery perforator flap where one perforator originated from the medial branch of the deep branch of the MPA and the other from the superficial branch of the MPA. Details of the patients are presented in Table 1.

**Table 1:** Summary of the patients receiving MPAP flap.

Case	Gender	Age	Side	Etiology	Flap Size (cm)	Origin of Perforator	Number of Lobulated Flaps	Flap Donor Site	Follow-Up (months)	Complication
1	M	57	right palm and finger web	Scar contracture	5×7.5	The medial branch of the deep branch of the MPA	2	skin graft	7	No
					4×4	The Superficial branch of the MPA				
2	M	58	volar of the left thumb	crush	2×3	The medial branch of the deep branch of the MPA	1	Primary closure	4	No
3	M	44	volar of the left thumb	crush	3.5×3.5	The medial branch of the deep branch of the MPA	1	Skin graft	8	No
4	F	37	volar of the index finger	crush	3.5×3.5	The medial branch of the deep branch of the MPA	1	Skin graft	3	No
5	M	57	volar of the left thumb	crush	3×3.5	The medial branch of the deep branch of the MPA	1	Skin graft	5	No
6	M	49	volar of the left thumb	crush	3×4	The medial branch of the deep branch of the MPA	1	Skin graft	6	No
7	M	47	right palm	Scar contracture	3×7	The medial branch of the deep branch of the MPA	1	Skin graft	8	No
8	M	44	Dorsal and volar of the index finger	crush	3×6	The medial branch of the deep branch of the MPA	1	Skin graft	6	No
9	M	11	volar of the middle finger and the ring finger	Scar contracture	1.5×5.5	The medial branch of the deep branch of the MPA	2	Skin graft	8	No
					1.5×5.0	The superficial branch of the MPA				
10	F	35	The left middle finger	Degloved injury	3×6	The medial branch of the deep branch of the MPA	2	Skin graft	5	No
					3×7	The superficial branch of the MPA				
11	M	45	dorsal of the left middle finger	crush	3×4	The medial branch of the deep branch of the MPA	1	Skin graft	6	No

### Anatomy and surgical technique

**Figure 1:**
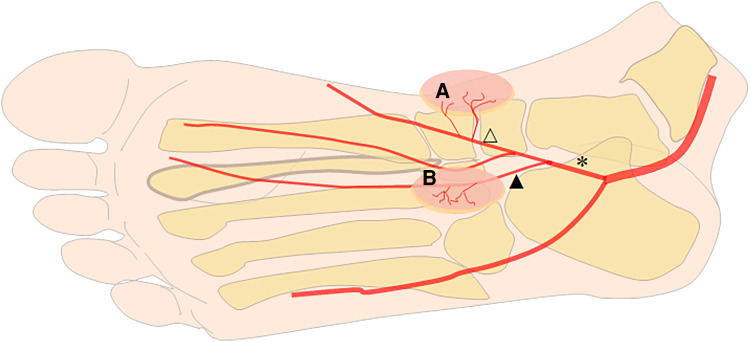
Schematic diagram of blood flow to the medial plantar artery perforator (MPAP) flap. A lobulated MPAP flap (**A** and **B**) originates from the medial branch of the deep branch of the MPA (blank triangle) and the superficial branch of the MPA (black triangle). Star: the medial plantar artery (MPA).

After the tarsal tunnel, the posterior tibial artery is divided into the medial plantar artery and the lateral plantar artery. After emerging from the posterior tibial artery, the medial plantar artery enters the compartment of the sole of the foot under the abductor hallucis muscle. Roughly at the joint level, the medial plantar artery is divided into superficial (lateral) and deep (medial) branches (15, 18, 20). The superficial branch of the MPA runs between the abductor hallucis and the flexor digitorum brevis and finally anastomoses with the first plantar metatarsal artery. After emerging from the MPA, several perforators directly issue from the superficial branch of the MPA and nourish the instep skin. These skin branches provide the basis for one of the lobulated MPAP flaps. The deep branch of the MPA lies on the medial side of the superficial branch of the MPA and is divided into the medial branch and the lateral branch. After division from the deep branch of the MPA, the medial branch courses from the plantar to the tubercle of the navicular bone. Then, the medial branch continues distally and constantly anastomoses with the first plantar metatarsal artery. The lateral branch penetrates even deeper into the planta pedis and terminates on the medial segment of the deep plantar arch. About 1 cm from the proximal of the navicular bone, several perforators originate from the medial branch of the deep branch of the MPA and supply the skin of the medial aspect of the foot (15, 18, 21, 22). These branches form the anatomical basis of the other of the lobulated MPAP flap (Figure 1).

To begin with, the surface outline of the MPA was drawn and the perforators from the medical branch of the deep and superficial branches of the MPA were detected and marked by Doppler probe. Also, patients were given a Doppler test to confirm the patency of the posterior tibial artery (PTA) and dorsalis pedis artery (DPA) in the pre-operative period.

After scar release or debridement of injured hands, the shape and the size of the defects were measured, and the territory of the flap centered on the perforators was drawn. Under tourniquet control, the first incision of the flap was made parallel to the abductor hallucis muscle, creating the medial border of the flap. When the flap was dissected laterally, several perforators that nourished the flap were found. Retrograde dissection was performed along the perforator to locate the medial branch of the deep division of the MPA, which is the truck of the perforator. Then, the lateral border of the flap was then incised and dissected. The distal of the medial branch of the deep division of MPA was ligated and the proximal continued to be dissected between the abductor hallucis and the medial skeletal margin of the foot. Retrograde dissection was performed along the medial branch of the deep division of the MPA to the truck of the MPA and the lateral branch of the deep division of the MPA was ligated (18). A similar operation was performed on the other flap, which was supplied by the perforators from the superficial branch of the MPA. Briefly, after the first incision of the flap was made parallel to the abductor hallucis muscle, perforators were identified between the abductor hallucis and the flexor digitorum brevis and traced to the truck of the MPA. All single-lobulated MPAP flaps were harvested from the perforators from the medial branch of the deep branch of the MPA. The perforator arteries are always small in the MPAP flap (around 0.6–0.8 mm) (16). The length of the vessel pedicle can reach approximately 3–5 cm without causing damage to the lateral plantar artery (Figure 1). Then, the flap was transferred to the defect of the hand. In all cases, arterial anastomoses were performed between the medial plantar artery and the available digital artery in the hand. Two concomitant veins of the flap were anastomosed to the dorsal veins. Venous grafts of the forearm may be used when the vein length is insufficient. The donor site was covered by a full-thickness skin graft or by primary closure.

Post-operatively, patients were maintained on strict bedrest with their hands fixed by plaster for 1 week. During this time, the color and temperature of the flap were checked periodically. Vascular emergent surgery would be performed when the flap color turned gray. After 1 week, the patients began to move out of bed and exercise. After 2 weeks, the plaster was removed and the fingers began to practice.

## Results

A total of 11 flaps were fixed in 11 patients. Of the 11 patients, 3 were fixed with two-lobulated medial plantar perforator artery flaps. The other 8 patients had single-lobulated MPAP flaps, which were harvested from the medial branch of the deep branch of the MPA. The size of the flaps ranged from 2×3 to 5×7.5 cm. Every flap contained 1–2 perforators, and the mean vascular pedicle length was 4.5 cm with a range of 4–6 cm. All flaps survived completely with no arterial or venous thrombosis. No emergency surgery for the vessel was required. The mean follow-up time was 6 months (ranging from 3 to 8 months). All flap wounds healed well. No finger ischemia or delayed wound healing occurred. During the follow-up period, the patients were satisfied with their appearance without any traces of flap plastic surgery. One donor site was closed primarily, and the others were covered with a full-thickness skin graft. No complaints from the donor site were reported from all patients. Details are given in Table 1.

### Case 1

**Figure 2:**
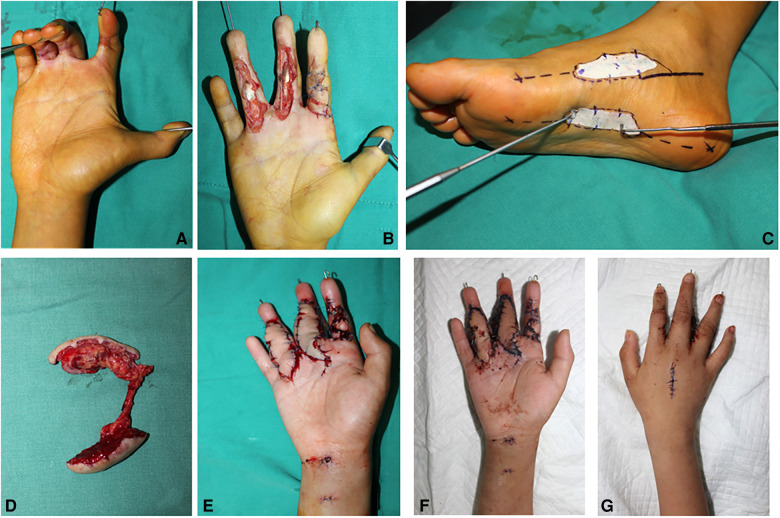
Case 1: Pre-operative view of a scar contracture of the finger (**A**). The defect of the finger following scar release (**B**). A two-lobulated MPAP flap (7×3 cm^2^ and 6×3 cm^2^) is marked (**C**). The two-lobulated MPAP flap is harvested (**D**). The MPAP flap is transferred to the defect in the finger (**E**). Appearance at 2 weeks of follow-up (**F,G**).

An 11-year-old boy suffered scalds on multiple fingers, which resulted in scar contracture and dysfunction (Figure 2A). The index finger straightened on account of “Z” incision. After scar release and joint fix by Kirschner wires, a 1.0×5.0 cm^2^ defect in the middle finger and a 1.0×4.5 cm^2^ defect in the ring finger were measured. The tendon and the digital nerve were exposed (Figure 2B). A two-lobulated medial plantar artery perforator flap (1.5×5.5 cm^2^ and 1.5×5.0 cm^2^) was designed and harvested from the ipsilateral foot (Figures 2C,D). Then, the flap was transferred to cover the defect in the hand. The artery of the flap was anastomosed with the ulnar digital artery of the middle finger. Venous anastomosis was performed between two concomitant veins and the dorsal veins. In this case, vein graft in the forearm was harvested to make up for the lack of the venous length of the flap (Figure 2E). A full-thickness skin graft from the ipsilateral thigh was used to close the donor sites. The flap survived and healed completely without any complications (Figures 2F,G). The skin in the donor site also survived and healed completely. In the 3rd week after surgery, the Kirschner wires were removed, and the finger began to practice. The appearance is found satisfactory after 6 months of follow-up.

### Case 2

**Figure 3:**
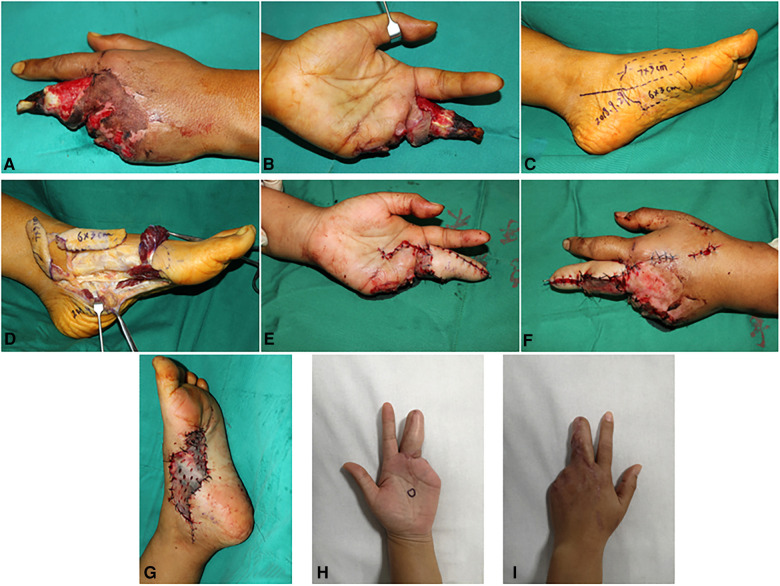
Case 2: Pre-operative view of the defect (**A,B**). A multipage MPAP flap (7×3 cm^2^ and 6×3 cm^2^) designed according to the size of the defect is marked (**C**). A two-lobulated MPAP flap is harvested (**D**). The MPAP flap is transferred to the defect in the finger (**E,F**). Full-thickness skin grafting is performed at the donor site (**G**). Final appearance at 6 months of follow-up (**H,I**).

A 35-year-old woman suffered a degloving injury on her left middle finger and lost her left ring finger and little finger (Figures 3A,B). After complete debridement, a two-lobulated medial plantar artery perforator flap (3×6 cm^2^ and 3×7 cm^2^) was designed and harvested from the ipsilateral foot to cover the middle finger (Figures 3C,D). The flap was transferred to restructure the middle finger (Figures 3E,F). The flap artery was anastomosed with the radial digital artery of the middle finger. Two concomitant veins were repaired to the dorsal veins. No vein graft in the forearm was performed. A full-thickness skin graft from the ipsilateral thigh was used to close the donor sites (Figure 3G). Both flaps survived completely and proved uneventful. The grafted skin in the donor site also survived and healed completely. During 3 months of follow-up, the patient did not complain of any discomfort and was satisfied with her appearance (Figures 3H,I).

### Case 3

**Figure 4:**
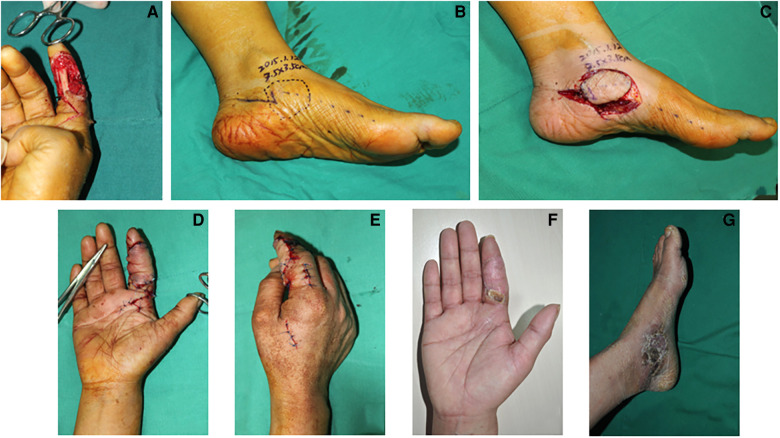
Case 3: The defect in the finger after debridement (**A**). An outline of an MPAP flap (3.5×3.5 cm^2^) based on the perforators of the medial branch of the deep branch of the MPA is drawn (**B**). The MPAP flap is harvested (**C**). The appearance of a finger with the MPAP flap reconstruction (**D,E**). Follow-up at 3-month post-operative period (**F**, finger and **G**, donor site).

A 35-year-old woman suffered from a crush injury resulting in a necrosis of the volar skin and exposure of the tendons and nerves in the index finger. After debridement, a defect of about 3.0×3.0 cm^2^ in the index finger was noted (Figure 4A). A 3.5×3.5 cm^2^ medial plantar artery perforator flap was designed and harvested from the medial side of the contralateral foot where the perforator was derived from the medial branch of the deep division of the MPA (Figures 4B,C). Then, the flap was transferred to cover the defect in the index finger. The flap artery was anastomosed to the superficial radial artery and the concomitant veins were repaired to the dorsal veins (Figures 4D,E). In this case, the medial branch of the deep branch of the MPA was anastomosed with the distal digital artery to bridge and reconstruct the blood flow of the finger. The donor site was closed by a full-thickness skin graft from the contralateral thigh. Both the map and the grafted skin in the donor site survived and healed completely. During 4 months of follow-up, the appearance of the index finger was satisfactory (Figures 4F,G).

## Discussion

Complex soft tissue defects of the finger induced by severe trauma are very common in clinical practice, which always lead to multiple fingers or site involvement, and the exposure of tendons, nerves, and bones. The reconstruction of soft tissue defect in the finger remains a challenge for surgeons. Many factors need to be considered for optimal surgical methods that can repair soft tissue defects in the fingers, such as durability, texture, size, sensation, function, aesthetics, and donor site morbidity. Although a number of methods have been reported, many severe soft tissue defect cases have not been covered (1, 23).

Traditional methods have been widely used and have achieved good repair results, including V-Y advancement flaps, cross-finger flaps, and digital artery island flaps (1, 7). However, there are obvious deficiencies that limit their application. V-Y advancement flaps have only one short advancement distance (7). Cross-finger flaps require a second operation to remove the pedicle (24). As to digital artery island flaps, surgeons have to sacrifice a main artery (1). What is more, traditional methods are limited in flap size and not suitable for moderate/large defects or multifinger defects (20). Distant pedicled flaps, such as radial forearm flaps, can be used for larger defects, but they have a limited pedicle transfer length and can result in longer immobilization and less durability (23). Free flaps are a good choice and can cover larger or multifinger defects, such as the groin flap and posterior interosseous flap. However, the bloat of free flaps, which requires another flap plastic surgery, and less durability also limit the application (21, 25). To solve these problems, the medial plantar artery perforator flap is an ideal option for the reconstruction of soft tissue defect in the fingers (4, 21).

The medial plantar flap harvested from the hairless non-weight-bearing medial plantar region of the foot sole and nourished by the medial plantar artery was first described by Shanahan and Gingrass in detail (26). Based on the two branches of the medial plantar artery, different types of flaps have also been reported. The blood supply of the instep flap (also called the medial plantar flap) is from the superficial branch of the medial plantar artery (13, 27). The medial pedis flap was nourished by the medial branch of the deep branch of the medial plantar artery (3). In 2007, Koshima et al. originally described the medial plantar artery perforator (MPAP) flap (14). Different from the instep flap or the medial pedis flap, the perforator of this flap directly arises from the proximal of the deep branch or the trunk of the medial plantar artery (15, 18). Since its introduction, the medial plantar flap has been widely used by surgeons for the reconstruction of the feet, ankles, knees, hands, and fingers. Compared with other flaps, many advantages have been noted for the reconstruction of soft tissue defect in the finger. (1) The flap size range is wide enough to make up for various defects (2×2 cm^2^∼12×8 cm^2^) (18, 27), whether it is the defect of the palm or of the digital. (2) The medial flap is thinner than other flaps. The finger, which received reconstruction by the medial flap, returned to its original good appearance without any need for performing a second plastic surgery (18). (3) There are two accompanying veins in the vascular pedicle of the flap. Even another subcutaneous vein in the flap can be made available. These veins can promote the venous return of the flap and reduce the incidence of congestion (3). (4) The color and texture of the medial plantar flap is similar to that of real human fingers. More durability can be achieved. (5) The recovery of protective sensation in the finger is good with or without neurorrhaphy since the medial plantar flap is thin (15). (6) The medial plantar flap is harvested from the non-weight-bearing medial plantar region and has minimal donor site morbidity. Therefore, it is reliable to use the medial plantar flap as a donor reconstruction of the finger.

In this research, we narrated our experience using the MPAP flap for the reconstruction of complex defects of the finger. All flaps survived completely with satisfactory appearance. No complications were observed. Although some studies have been reported about the reconstruction of the finger by the MPAP flap, our research has some novelties. Perforators of the MPAP flap in our cases were derived from the medial branch of the deep division of the MPA or the superficial branch of the MPA, but those in prior reports come from the proximal of the deep branch or the trunk of the medial plantar artery. What is more, the lobulated flaps are designed to accommodate complex soft tissue defects involving multi fingers or multi sites. The MPAP flaps in prior reports were used for single fingers or sites and achieved good recovery (4, 15, 21).

Based on the two branches of the medial plantar artery, the lobulated MPAP flap has an advantage in terms of repairing complex defects in fingers. A combined medial pedis flap and instep flap to restore the soft tissue defect of finger has been reported (20, 28). However, both the instep flap and the medial pedis flap are classified as a pedicle fasciocutaneous flap and are different from the perforator flap. With the introduction of the perforator concept and the development of a free-style free perforator flap, more and more free perforator flaps have been applied in clinical cases (29, 30). Combining the perforator concept and the anatomy of the medial plantar artery, it is possible to design a lobulated MPAP flap (18). Compared with the instep flap or the medial pedis flap, the MPAP flap in this article has thinner thickness and a better appearance.

While the reconstruction of soft tissues in fingers is achieved, the morbidity of the donor site cannot be ignored. Currently, many strategies have been applied in the donor site, including direct closure, split-thickness skin grafting (STSG), full-thickness skin grafting (FTSG), pre-operative tissue expansion, and dermal substitutes, and so on (2, 31). Due to the small movement of the foot skin, the donor site of the MPAP flap usually cannot be directly closed. In our report, full-thickness skin grafts were designed for 10 of 11 patients. Although STSG or FTSG has been widely used in skin defects, disadvantages (scar contracture, unsatisfactory appearance, and poor skin elasticity) are also obvious (31). In response, recent studies have revealed that dermal substitutes could help reduce these disadvantages (2, 31). Watfa et al. showed that the combination of an STSG with MatriDerm substantially decreased donor site morbidity and achieved better repair than FTSG (31). So, dermal substitutes are also recommended for future work. Although the MPAP flap is an ideal option for the coverage of finger tissue loss, several disadvantages should be noticed. (1) The perforator of the MPAP flap is small and variable. A sound anatomical knowledge and surgical experience are required for performing this surgery, which limits the application of this flap. (2) We need more time to confirm and dissect perforators. As a result, the overall duration of the operation will be extended. Therefore, many factors should be considered for the reconstruction of finger defects, including durability, size, protective sensation, morbidity, and procedure. Recently, di Summa et al. described that the DBAp (distal brachial artery perforator) flap was used to reconstruct complex digit defects and presented a good result (32). The DBAp flap can be designed as a neurotized flap or composite flap, containing skin and bone to meet the requirements of finger composite defects (32). Different from the DBAp flap, the MPAP flap can be harvested as a lobulated flap with a certain length of the vascular pedicle to meet multiple finger defects. The medial plantar flap with similar skin color and texture provides more durability for the fingers. The MPAP flap is an ideal option for repairing complex finger defects, especially multifinger soft tissue defects.

## Conclusion

In our study, we reported our experience in using the MPAP flap for the reconstruction of complex finger defects. All flaps survived completely with satisfactory appearance. No complications were observed. In summary, the MPAP flap is a reliable and acceptable option for the reconstruction of complex soft tissue defects in the finger. Depending on the two branches of the medial plantar artery, the lobulated MPAP flap is promising for soft tissue defect reconstruction involving multiple fingers or sites.

## Data Availability

The original contributions presented in the study are included in the article/Supplementary Material; further inquiries can be directed to the corresponding author/s.
